# Diffuse Large B Cell Lymphoma Raising Suspicion for an Infection: A Case Report

**DOI:** 10.7759/cureus.34750

**Published:** 2023-02-07

**Authors:** Tyrell Daniel, Ryan Sweeney, Aaron Haag, Suzanne Morrissey

**Affiliations:** 1 Medicine Institute, Allegheny Health Network, Pittsburgh, USA; 2 Department of Gastroenterology and Hepatology, Allegheny Health Network, Pittsburgh, USA

**Keywords:** dlbcl, liver mass, r-chop therapy, actinomycosis, diffuse large b cell lymphoma, primary hepatic lymphoma

## Abstract

Newly discovered liver lesions have a broad differential ranging from malignancy to infection. While tissue biopsy is the gold standard diagnostic modality, imaging can also aid in diagnosis. Hepatocellular carcinoma (HCC) can be diagnosed via imaging alone; however, masses suspicious for infection ultimately require biopsy and culture. We report a case of a 72-year-old male who presented with subjective fever, nausea, decreased appetite, dark urine, elevated liver function tests, and CT evidence of an exophytic liver mass. Differentials included infections such as hepatobiliary actinomycosis, abscess, solid malignancy, or lymphoma. Obtaining a definitive diagnosis with tissue biopsy endoscopically and percutaneously was quite difficult due to the location of the lesion around the porta hepatis. Subsequent laparoscopic biopsy revealed diffuse large B cell lymphoma (DLBCL).

## Introduction

Primary hepatic lymphoma (PHL) is a rare presentation of non-Hodgkin’s lymphoma (NHL). Typically, NHL involves the lymph nodes or spleen and only secondarily involves the liver via metastases [1-2]. In a study assessing the utility of surgical procedures for staging, 43% of the 170 patients evaluated were found to have documented liver metastases [1]. However, PHL represents only 0.016% of all newly diagnosed NHL cases [2]. Diagnostic criteria for PHL as defined by Lei in 1998 include symptoms related to liver involvement, absence of lymph node and bone marrow involvement, and absence of abnormal cells in the peripheral smear [3]. Histopathological findings are needed to differentiate PHL as it may be diffuse large B cell lymphoma (DLBCL) or T cell lymphoma and, in some cases, even non-B non-T cell lymphoma [1]. DLBCL is the most commonly observed PHL [1]. The common presenting symptoms include constitutional B symptoms, jaundice, abdominal discomfort, nausea, and vomiting [4]. These symptoms of PHL may raise suspicion for infection; hence, a thorough history, physical examination, biochemical markers, and imaging are needed to rule out infectious etiology. There are well-known mimickers of liver malignancy or lymphomas such as liver abscess, hepatic actinomycosis, and fungal infections [5-7]. Hence, further testing with contrasted imaging of CT or MRI is indicated. However, if acute kidney injury is present, some nephrologists may opt to defer the use of contrasted imaging to prevent the worsening of renal function and prevent gadolinium-induced nephrogenic systemic fibrosis when glomerular filtration rate (GFR) is <30. Hence, tissue culture and biopsy may be needed for a definitive diagnosis. In light of this, we discuss a case that presented a clinical dilemma as to whether a hepatic mass was hepatocellular carcinoma (HCC), lymphoma, or an abscess.

## Case presentation

**Figure 1:**
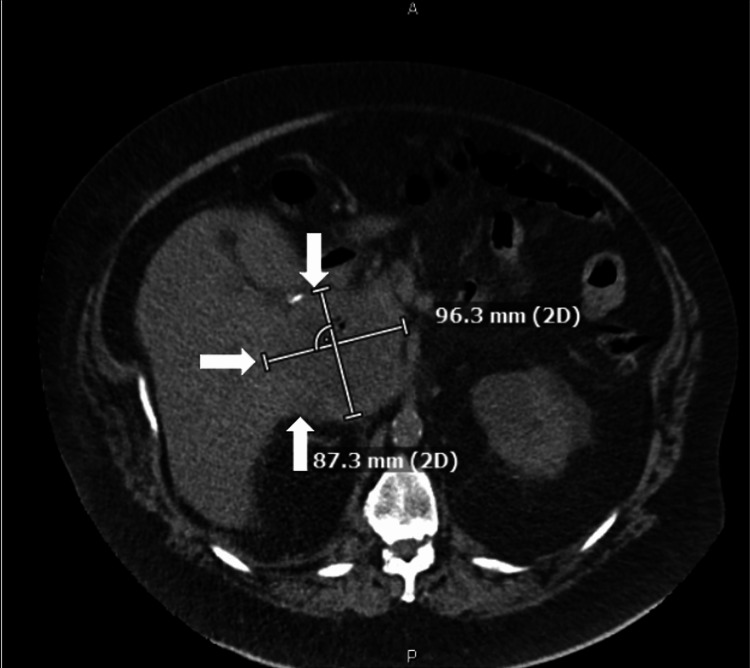
CT of the abdomen/pelvis without contrast revealing a 9.63 x 8.73 cm mass (indicated by arrows) involving the porta hepatis CT: computed tomography

A 72-year-old male with a past medical history of morbid obesity, controlled non-insulin-dependent diabetes mellitus, coronary artery disease, chronic kidney disease stage 3, and laparoscopic cholecystectomy presented to an outside hospital with a chief complaint of subjective fever, nausea, dark urine, and decreased appetite. On exam, he was found to be jaundiced with abdominal distention and diffuse abdominal tenderness. His labs were significant for alkaline phosphatase of 603 U/L (normal range: 40-129 U/L), aspartate aminotransferase of 187 U/L (normal range: 0-40 U/L), alanine transaminase of 184 U/L (normal range: 0-41 U/L), total bilirubin of 11 mg/dL (normal range: 0-1.2 mg/dL), creatinine of 4.5 mg/dL (normal range: 0.70-1.20 mg/dL) with a baseline of 1.6 mg/dL, and GFR of 16. CT of the abdomen/pelvis without contrast was concerning for a developing abscess or contained duodenal perforation vs. exophytic liver lesion (Figure 1). The patient was then transferred to our tertiary care center on the same day for further evaluation by the gastroenterology and surgical teams.

**Figure 2:**
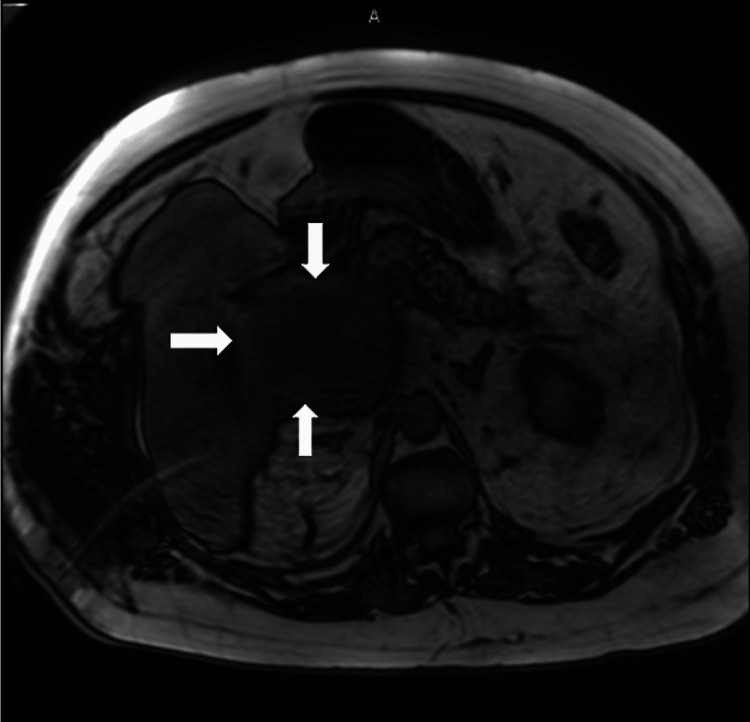
MRCP without contrast with a T2 image revealing an isointense or hyperintense density (indicated by arrows) adjacent to the liver parenchyma, concerning for a solid tumor mass vs. abscess MRCP: magnetic resonance cholangiopancreatography

On presentation at our institution, the patient's temperature was 36.4 °C, blood pressure was 111/46 mmHg, pulse was 67, respiration rate was 18 breaths/minute, and he was saturating 92% on room air; the physical exam revealed a jaundiced male who looked his stated age with abdominal distention and diffuse abdominal tenderness. Laboratory results noted alkaline phosphatase of 604 U/L, aspartate aminotransferase of 187 U/L, alanine transaminase of 174 U/L, total bilirubin of 10.2 mg/dL, creatinine of 4.52 mg/dL, and GFR of 16. Carcinoembryonic antigen (CEA) and alpha-fetoprotein (AFP) were negative. Cancer antigen 19-9 (CA 19-9) was mildly elevated at 76 U/ml (normal: <55 U/ml). The patient was started on intravenous cefepime 1 g every 12 hours (renal dosed) and metronidazole 500 mg every 12 hours due to suspected infection. Gastroenterology was consulted and recommended an MRI abdomen and magnetic resonance cholangiopancreatography (MRCP) without contrast. The MRCP confirmed the presence of the lesion (Figure 2).

However, based on the appearance, the diagnosis now favored malignancy more severe than an infection. Notably, the spleen was not enlarged and there was no abdominal lymphadenopathy. Endoscopic retrograde cholangiopancreatography (ERCP) was performed on hospital day three. The endoscopist noted the perihilar liver mass but deemed it not feasible to biopsy via endoscopic ultrasound (EUS) due to poor positioning. ERCP revealed biliary dilation with pus in the biliary duct, thereby not ruling out an infection as the cause of the liver lesion. Infection remained on the differential due to these findings as it was unclear if this was a superimposed infection upon malignancy or not. Hepatobiliary actinomycosis was now on the differential since it is notorious for mimicking hepatobiliary malignancy with a mass-like appearance on imaging [5-7].

Further testing was necessary with MRI with contrast to rule out abscess but, unfortunately, the course was complicated by his acute kidney injury, and hence Nephrology was consulted for their recommendation on contrast administration given his decreased GFR but with adequate urine output of about 1.5 liters per day. Nephrology suggested pre-renal as the most likely cause of his kidney injury because the patient had a prolonged decreased oral intake, and CT had noted no post-renal obstructive processes. Complement levels, cryoglobulin, antineutrophil cytoplasmic antibodies (ANCA) panel, and anti-glomerular basement membrane antibodies tests were ordered and were negative. Nephrology conducted urine microscopy, which noted granular muddy brown casts notable for acute tubular necrosis, and decided to optimize his renal function by administering lactated Ringer's infusion to perfuse his kidneys to avoid the need for dialysis. Given his tenuous renal function, the interdisciplinary team opted to avoid contrasted studies and planned to obtain a confirmatory biopsy and culture of the liver lesion. Biopsy was attempted twice without success, once with fine needle biopsy via EUS, which was non-diagnostic with negative stains for infection. Another attempt at biopsy was made via a percutaneous CT-guided approach; however, this was aborted due to the patient’s newly developed intolerance for lying flat with profound dyspnea and hypoxia, which was believed to be secondary to hypervolemia after the administration of intravenous fluids. Intravenous fluids were stopped given the improvement of his renal function, Nephrology gave the approval to use the gadolinium contrasted study if needed as his GFR was now 60. However, the surgical team along with the patient decided to do an outpatient laparoscopic biopsy for a definitive diagnosis. Sixteen days after his initial transfer, the patient was discharged to a rehabilitation facility to improve his physical conditioning on intravenous ampicillin-sulbactam 3 g every six hours for possible intra-abdominal infection, with close infectious disease follow-up as a definitive antibiotic timeline could not be established until a definitive tissue biopsy was obtained. 

Eleven days after rehabilitation, a laparoscopic tissue biopsy was collected, which revealed DLBCL, germinal center phenotype, with a high proliferation fraction at 95%. Immunohistochemistry was positive for CD20, CD79a, CD30, MUM1, CD10, BCL2, and BCL6 and negative for ALK and Cyclin D1. Fluorescent in situ hybridization (FISH) was notable for BCL2 rearrangements and negative for MYC, IGH, and BCL2 rearrangements. The liver biopsy also noted cholestatic changes consistent with biliary obstruction and steatohepatitis. Given the diagnosis of DLBCL, a positron emission tomography (PET) scan was ordered, which demonstrated a hypermetabolic right upper quadrant mass compatible with known malignancy. Chemotherapy with R-CHOP (Rituximab, cyclophosphamide, doxorubicin, vincristine, and prednisone) regimen was initiated one month after discharge. Unfortunately, the patient died one month after commencing therapy.

## Discussion

As PHL represents an exceedingly rare diagnosis, the differential diagnosis for a patient presenting with a solitary liver lesion is broad and includes HCC, lymphoma, metastatic liver disease, and infectious and benign etiologies. A biopsy is required to confirm the diagnosis. Our patient presented with non-descript symptoms including abdominal pain, nausea, dark urine, and decreased appetite. Laboratory evaluation revealed elevated liver function tests, prompting a CT scan, which revealed findings of a liver mass vs. a developing abscess. Following multiple non-diagnostic biopsy attempts, with a liver mass found on imaging, and ERCP findings of biliary dilation and pus in the biliary tract, the patient was discharged with a presumed diagnosis of hepatobiliary actinomycosis based on previous case reports that had depicted similar characteristics that mimic hepatobiliary cancer [8]. Actinomyces, a Gram-positive anaerobic bacteria, represents a rare cause of abdominopelvic abscesses [9]. The postulation of the sequence of events was as follows: a biliary stricture that resulted in chronic stasis followed by the formation of hepatic abscess/ascending cholangitis. Hence, he was discharged on outpatient intravenous antibiotics with plans for a definitive biopsy as an outpatient. Ultimately, the gold standard tissue biopsy via laparoscopic approach revealed DLBCL, and the patient was initiated on chemotherapy.

The etiology of PHL remains unclear; however, there appears to be an association with chronic infections including hepatitis C, hepatitis B, hepatitis E, HIV, and cytomegalovirus, in addition to autoimmune disease [10,11]. Our patient notably had no history of autoimmune disease and tested negative for hepatitis B; however, he was not tested for any of the other associated infections. The most common PHL is DLBCL, accounting for 46-96% of cases. The mean age at diagnosis is 50-62 years and it most commonly affects men [12]. The second most common etiology of PHL is extranodal marginal zone lymphoma of mucosa-associated lymphoid tissue [12].

Management of PHL is challenging given the rarity of the disease and the lack of prospective studies to develop treatment guidelines. A case series assessing survival outcomes following surgical resection of eight patients demonstrated a median survival of 23 months [13]. In a retrospective cohort review of 24 patients, 83.3% of patients achieved complete remission following combination chemotherapy with a five-year overall survival rate of 83.1% [14]. Common chemotherapy regimens include CHOP, achieving a complete response in 9/13 patients in a retrospective review [14]. Unfortunately, our patient died approximately one month after initiating treatment with R-CHOP.

## Conclusions

This presentation of PHL involved a clinical dilemma about whether the liver mass seen on imaging was truly a solid malignancy, lymphoma, or infection. The patient's acute kidney injury and the interdisciplinary team's desire to preserve renal function to avoid dialysis initially precluded the use of contrast CT or MRI, which may have improved the likelihood of ruling out an infection. Despite the absence of contrasted imaging, the clinical picture was also skewed when the ERCP noted pus in the biliary tract, which may point to an infection. Therefore, this case highlights the importance of tissue biopsy and culture to achieve a definitive diagnosis of liver lesions. However, adequate tissue biopsy via an endoscopic approach for some lesions may be difficult due to their location. Additionally, a patient’s inability to lie flat with stable vital signs was also demonstrated as a limiting factor for performing a successful CT-guided biopsy. Fortunately, the diagnosis of primary hepatic DLBCL was made via the laparoscopic approach, and treatment was commenced with R-CHOP. Due to the rarity of liver DLBCL, further retrospective analyses and case series are needed to better understand the prognostic factors and response to chemotherapy to improve patient outcomes.
